# The PROMISE study protocol: a multicenter prospective study of process optimization with interdisciplinary and cross-sectoral care for German patients receiving hip and knee endoprostheses

**DOI:** 10.1080/17453674.2020.1853927

**Published:** 2020-12-10

**Authors:** Ulrich Betz, Laura Langanki, Florian Heid, Jan Spielberger, Lukas Schollenberger, Kai Kronfeld, Matthias Büttner, Britta Büchler, Markus Goldhofer, Lukas Eckhard, Philipp Drees, the PROMISE GROUP

**Affiliations:** a Institute of Physical Therapy, Prevention and Rehabilitation, University Medical Center of the Johannes Gutenberg University, Mainz;; b Department of Anaesthesiology, University Medical Center of the Johannes Gutenberg University, Mainz;; c Department of Anaesthesiology and Intensive Care Medicine, St Josef Hospital, Vienna, Austria;; d Interdisciplinary Center for Clinical Trials, University Medical Center of the Johannes Gutenberg University, Mainz;;; e Institute of Medical Biostatistics, Epidemiology and Informatics, University Medical Center of the Johannes Gutenberg University, Mainz;; f Department of Trauma and Orthopedic Surgery, Hunsrück Hospital Kreuznacher Diakonie, Simmern/Hunsrück;; g Department of Orthopedics and Traumatology, University Medical Center of the Johannes Gutenberg University, Mainz, Germany

## Abstract

Background and purpose — Knee and hip replacement are common and increasing procedures, and an optimized care process that could be implemented in different settings would be useful. The PROMISE trial investigates whether a new care process works equally in different German settings and how the results compare with current non-standardized care.

Patients and methods — This multi-center prospective mixed-method study includes 2,000 German patients receiving arthritis-related hip or knee endoprostheses. An interdisciplinary and cross-sectoral care process was developed and implemented in 3 German hospitals with different levels of care, and corresponding rehabilitation centers were included to bridge the gap after acute care.

Duration and outcome — The PROMISE trial recruited patients between May 2018 and March 2020. Follow-up will end in February 2021. Assessments are performed at: examination on clinical indication, 1 week before surgery, on the day of surgery, at the end of hospitalization, end of the rehabilitation program, and 3 months, 6 months, and 12 months after surgery. Outcomes include patient-reported outcomes, medical examination findings, and routinely collected data regarding the surgery and complications. Guideline-based interviews are conducted with selected patients and care partners. The primary endpoint is the presence of chronic pain at 12 months after surgery. Secondary endpoints are the number of recognized pre-existing conditions, physical activity at 12 months after surgery, use of medical services, quality of life, and interactions between care partners.

Trial registration — The trial is registered with the German Clinical Trials Register (https://www.drks.de; DRKS00013972; March 23, 2018).

With demographic changes joint replacement is becoming one of the most frequently performed surgeries. In Germany, approximately 175,000 total hip arthroplasties (THA) (IQTIG 2018a) and 148,000 total knee arthroplasties (TKA) (IQTIG 2018b) are performed each year.

These replacement procedures are associated with various risks and complications, including infection and thrombosis, as well as a considerable financial burden. Moreover, 7% to 23% of patients with THA and 10% to 34% of patients with TKA report an unfavorable long-term outcome (Beswick et al. 2012). Due to the high number of these interventions, clinical, patient-centered, and economic results might be improved by even minor advancements in the treatment process. In Germany no generally applicable evidence-based treatment standard has yet been developed. Thus, the current care system does not achieve its full potential, including the cost–benefit ratio.

The PROMISE trial aims to improve the care process in Germany based on the principles of the Enhanced Recovery after Surgery Society (ERAS). An ERAS path is defined to optimally prepare the patient for an intervention with minimized stress and stress reactions, maintained homeostasis, and avoiding catabolism that leads to loss of protein, muscle strength, and cellular dysfunction (Ljungqvist 2012). The ERAS approach has been applied to numerous elective procedures and provides approximately 30–50% reductions in the complication rate and length of stay (Ljungqvist et al. 2017). Because of a call for certified intersectoral centers to improve the quality of care (IGES Institut 2016), the PROMISE study involves 3 hospitals with different levels of care, including all required departments, as well as 5 inpatient and outpatient rehabilitation centers. Standard operating procedures defined by an interdisciplinary panel, and a central database for continuous evaluations, audits, and improvements (Ljungqvist et al. 2017) aim to minimize loss of potential effectiveness due to insufficient coordination, lack of common therapeutic goals, incomplete information flow, and separate data collection.

## ^Patients and methods^

### ^Study design^

The PROMISE trial is a prospective multi-center mixed-method study, including patients with an indication to undergo surgery (THA or TKA) at 3 German hospitals (a regional hospital, an orthopedic-specialized hospital, and a tertiary referral university hospital). The PROMISE process involves standardized indication criteria for intervention (Schmitt et al. 2017), preoperative screening for psychological (Gylvin et al. 2016) and geriatric risk factors (Gronewold et al. 2017), blood management (Vaglio et al. 2016), preoperative patient education (Edwards et al. 2017), no preoperative fasting (Smith et al. 2011), postoperative nausea and vomiting prophylaxis (De Oliveira et al. 2013), maximum soft-tissue-sparing techniques (Ljungqvist et al. 2017), intraoperative bleeding and swelling management (Guler et al. 2016, Nielsen et al. 2016), avoidance of suction drainage (Kelly et al. 2014), bladder catheters (Huang et al. 2015), and intravenous catheters (Sharma et al. 2010), local infiltration analgesia (Yun et al. 2015), multimodal oral pain therapy (Khan et al. 2014), starting rehabilitation on the day of surgery (Okamoto et al. 2016), functional discharge criteria (Hansen 2017), and intensified rehabilitation (DRV 2016). All patients are followed for up to 12 months after surgery. Data are collected by medical staff at the time of examination on indication, during patient education, at surgery, at hospital discharge, at the end of rehabilitation, and at 12 months after surgery. Patient-reported outcome measurements are obtained before surgery, at hospital discharge, and at 3 months, 6 months, and 12 months after surgery. Regarding secondary endpoints, a control group will be used consisting of patients from a German health insurance database. Patients will be matched according to age, sex, and diagnosis. To limit the number of survey-based instruments and patient burden, we conducted guideline-based interviews with 10 randomly selected patients to identify aspects that are difficult to capture using a quantitative approach. Interviews have been also conducted with different care partners (e.g., physicians and physiotherapists) to identify and address potential burdens.

### ^Study subjects and eligibility criteria^

Patients indicated for joint replacement due to arthritis of the hip or knee are eligible for study participation. Patients are enrolled if they have met standardized criteria for surgery (Schmitt et al. 2017) and if they are able to understand the nature and extent of the study.

*Exclusion criteria are:* life expectancy less than 1 year (e.g., advanced cancer), any conditions that might preclude elective surgical intervention, and medical or psychological factors that would prevent them from participating or providing informed written consent.

### ^Patient withdrawal^

Patients can withdraw their consent without giving reasons at any time during the trial without disadvantage. No additional data will be collected after that point, although any existing data will remain in the study database. Patients who withdraw are not replaced.

### ^Intervention^

**Figure 1.**
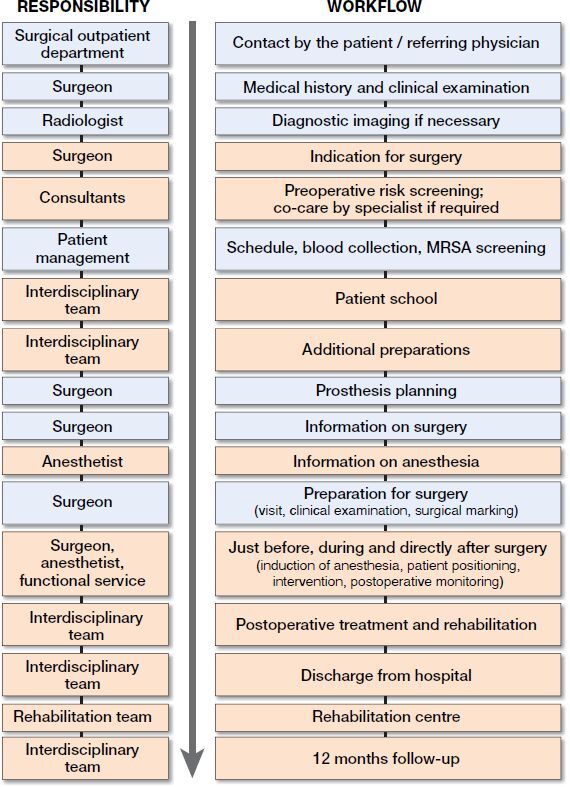
Schematic representation of the treatment process. PROMISE-specific procedures are shown in the red boxes. MRSA: methicillin-resistant *Staphylococcus aureus*.

The optimized PROMISE care process consists of interrelated measures (Figure 1). The ERAS Society guidelines (Wainwright et al. 2019) are evidence-based, although we complement those guidelines with non-evidence-based measures to avoid unnecessarily restricting the process optimization. These non-evidence-based measures are derived from a basic understanding of process optimization to avoid stress and promote activity.

### ^Indication for surgery^

The indication for surgery requires appropriate radiological findings, intolerable pain or suffering, restriction of activity, and exhaustion of nonoperative options (Schmitt et al. 2017). The patients define their activity or participation goals throughout the process.

### ^Preoperative screening for concomitant previous conditions^

Anemia is identified using the laboratory values for hemoglobin, ferritin, transferrin saturation, blood cell counts, red blood cell morphology, glutamic pyruvic transaminase, glutamic oxaloacetic transaminase, bilirubin, C-reactive protein, and reticulocytes. If necessary, iron is administered (orally or intravenously). Screening is performed to identify seniors at risk (ISAR screening) and psychosomatic risk (Patient Health Questionnaire-4 [PHQ-4], Oslo Social Support Scale [OSSS], Somatic Symptom Disorder [SSD], Life-Orientation-Tests [LOT-R]). Preoperative screening for thrombosis and bleeding risk is based on previous deep vein thrombosis or pulmonary embolism, known coagulation disorder/thrombophilia, venous thromboembolism in immediate relatives, previous severe bleeding, cancer within the last 5 years, and varicose veins. Specialized co-care is permitted if required.

### ^Patient education^

Patients are educated preoperatively by all involved professional groups. This process also involves discussing the patien’s tasks and co-responsibilities. Patients are also motivated to find a personal coach who supports the patient’s active role in the care process.

### ^Additional preparations^

Preoperative preparations include providing the patient with crutches and guidance regarding walking, using stairs, as well as instructions regarding preoperative fitness training. Patients also receive counseling regarding the rehabilitation options and programs (outpatient or inpatient).

### ^Immediately before surgery^

Medication for anxiolysis is avoided if possible. A light meal can be consumed up to 6 hours, and small amounts of clear liquids can be drunk up to 2 hours before anesthesia induction. Etoricoxib (90 mg) is administered 90 minutes and antibiotic (cefazolin 2 g i.v.) 30 minutes before the skin incision, followed by 1 g of tranexamic acid to reduce blood loss.

### ^During surgery^

General anesthesia and spinal anesthesia can be used. A single intraoperative steroid injection (e.g., 20 mg of dexamethasone or 125 mg of methylprednisolone) is used to attenuate the surgical stress response, improve analgesia, and reduce postoperative nausea and vomiting. The surgical technique aims to minimize trauma using the smallest possible incisions and natural muscle gaps. To prevent dislocation, the implanted THA must tolerate at least 30° internal rotation at 90° flexion and at least 70° external rotation in 0° extension. Local infiltration analgesia involves administration of ropivacaine (0.2%, 150 mL) with adrenaline (0.5mg/150mL) and an additional 50 mL of plain ropivacaine (0.2%) subcutaneously. Use of suction drainage and bladder/intravenous catheters is avoided whenever possible.

### ^Rehabilitation^

There are generally no restrictions regarding load bearing and range of motion. The patient begins independently leaving their bed and walking with crutches on the day of surgery, with support from the nursing staff or physiotherapist as necessary to achieve independent basic functions (changing position, personal hygiene, dressing and undressing, and eating at a table). Thromboprophylaxis is performed using low molecular weight heparins in weight- and risk-adapted dosage starting on the first postoperative day. In compliance with national guidelines thromboprophylaxis is continued for 35 (THA) or 14 (TKA) days postoperatively. Opioid use is minimized, and basic medication is used during days 3–8 (etoricoxib at 90 mg orally in the morning and metamizole 4 x 1 g). After day 8, the only medication is etoricoxib (90 mg orally in the morning). The patient is discharged to home or a rehabilitation facility when the wound is dry, pain is tolerable, and they can independently perform basic functions (change position, personal hygiene, dressing and undressing, walking > 150 m, and climb 10 steps). Patients who are discharged home also complete rehabilitation programs that comply with the rehabilitation therapy standards of the German Pension Insurance (DRV 2016).

### ^Endpoints^

#### Primary endpoint

The primary endpoint is presence of chronic pain at 12 months after surgery, evaluated with the pain subscore from the Western Ontario and McMaster Universities Osteoarthritis Index (WOMAC). Approximately 9% of patients report pain at 12 months after surgery (Beswick et al. 2012) and the evaluation is planned using a 2-sided binomial test. The WOMAC is calculated using the hip osteoarthritis outcome score (HOOS) (Klässbo et al. 2003) or the knee osteoarthritis outcome score (KOOS) (Roos et al. 2003).

#### Secondary endpoints

The secondary endpoints are number of recognized pre-existing conditions (as proxy we use pre-existing anemia) at baseline, physical activity at 12 months after surgery, use of medical services during the 12 months after surgery, quality of life at 12 months after surgery, and interactions between care partners during the 12 months after surgery. Physical activity is evaluated using the HOOS/KOOS subscore HOOS-PS/KOOS-PS (physical function short form) for THA/TKA cases, with data collected before the surgery and at 3 months, 6 months, and 12 months after the operation. Quality of life at 12 months after surgery is assessed using patient-reported scores from the EQ-5D-5L (Conner-Spady et al. 2015) and the EQ-5D visual analogue scale (VAS) on a 0–100 numeric scale. This data is also collected before surgery and at 3 months, 6 months, and 12 months after surgery.

Type and frequency of use of medical service during the 12 months after surgery is evaluated using a questionnaire. The results will be compared with results from a control group of patients from a German health insurance database who are matched according to age, sex, and diagnosis/treatment history. Statistical power for those analyses will be improved by matching 1 PROMISE participant to 4 control individuals. Cost analyses will also be performed to compare the PROMISE and control groups, to identify potential savings. Interactions between care partners during the first 12 months after surgery are also considered to determine whether a stable plateau is reached in the number of intersectoral interactions for each patient. The interviews will be evaluated according to the qualitative content analysis described by Mayring (2000).

### ^Data collection^

Data is collected using an electronic case report form, and the data types, collection processes, and entry processes were jointly developed by the PROMISE group to ensure all participating institutions and functional areas are working to the same standard.

Data is recorded and stored pseudonymized in a central electronic database at a specialized interdisciplinary center. During the trial, the investigators and trial site staff receive system documentation, training, and support for the use of the database. Time-point specific questionnaires and forms are completed by the patient, or the attending physician/physiotherapist, or both.

Data collection points (Table) are:

1. baseline visit,
2. 1 week before surgery,
3. day of surgery,
4. hospital discharge,
5. rehabilitation center discharge,
6. 3 months (±2 weeks) after hospital discharge,
7. 6 months (±2 weeks) after hospital discharge,
8. 12 months (±2 weeks) after hospital discharge,
9. and 12 months (±4 weeks) after hospitalization (clinical follow-up).

Baseline data includes age, sex, comorbidities, initial diagnosis according to International Classification of Diseases (version 10), and socioeconomic data. The other collected data includes:

- hip disability (HOOS),
- knee disability (KOOS),
- patient health quality (PHQ-4/SSD),
- quality of life (EQ-5D-5L),
- social support (Oslo Social Support Scale [OSSS]),
- personal expectations (Hospital for Special Surgery [HSS] score),
- rehabilitation success (Staffelstein score),
- and optimism/pessimism (Life Orientation Test-Revised [LOT-R]).

### ^Data quality assurance^

On-site qualified study nurses ensure that all personnel understand the trial and follow its protocol, including adhering to the standardized surgery and postoperative procedures. Surgeries are performed at centers that are qualified for the Enhanced Recovery process. A steering committee supervises the trial progress and provides guidance to all participating treatment groups.

Furthermore, the electronic database includes an interface to import quality-assured data that is sent by the participating hospitals to the Institute for Quality and Patient Safety.

### ^Estimated sample size and power^

The estimated sample size is based on the primary endpoint (chronic pain at 12 months), which will be evaluated using a 2-sided binomial test. A previous report has indicated that approximately 9% of patients report chronic pain after 1 year (Beswick et al. 2012). Thus, 1,900 patients are required to detect a 20% reduction (i.e., from 9% to 7.2%) based on an α level of 5% and power of 80%. Assuming 5% of patients will be lost to follow-up, the target sample size is 2,000 participants. This sample size also provides power of > 80% for analyzing the 2 confirmatory secondary endpoints (number of anemia cases and physical activity after 1 year; values selected from the literature), which will be analyzed only if a significant improvement is observed in the primary endpoint.

The secondary analyses are based on non-inferiority tests and previously reported values. The number of anemia cases will be tested to determine whether it is ≤ 2% below the expected reference value of 15.5% for Central Europe (McLean et al. 2009). Physical activity will be tested using the HOOS-PS/KOOS-PS to determine whether the patients achieve at least the minimum clinically important improvement (23 points) (Paulsen et al. 2014). To comply with the α-error, confirmatory testing for these 2 secondary endpoints will be performed only if statistically significant improvement is observed for the primary endpoint. To evaluate the achievement of a plateau in the number of interactions between care partners, the implementation period is divided into 3 intervals. The number of interactions in intervals II and III will be tested for equality. The criterion for equality will be based on the difference between the 1st and 2nd intervals. Poisson models will be used to compare the numbers of healthcare interactions between PROMISE participants and the control group, with sub-analyses according to different cost categories (e.g., hospital stays vs. general practitioner visits). Factors that potentially influence healthcare utilization, interactions, and quality of life will be evaluated using hierarchical regression models with care partners considered as clusters.

### ^Ethics, registration, funding, conflicts of interests, and result presentation^

The trial is conducted in accordance with the latest versions of the Declaration of Helsinki, Good Epidemiological Practice, and local regulatory requirements, including the German Federal Data Protection Act (Bundesdatenschutzgesetz). The protocol was approved by the ethics committees of Rhineland-Palatinate [837.533.17 (11367)], Baden-Wuerttemberg [B-F-2018-042], and Hessen [MC 84/2018]. The protocol is registered with the German Clinical Trials Register (DRKS00013972). Written informed consent is obtained from all patients before enrolment.

The trial is supported by a grant from the Federal Joint Committee (01NVF16015). None of the authors declare any conflicts of interest. Results will be presented at congresses and published in a peer-reviewed medical journal according to the guidelines of the International Committee of Medical Journal Editors.

### ^Study start and duration^

The 1st patient was recruited in May 2018, the last patient was recruited in March 2020, and follow-up will end in February 2021.

## ^Discussion^

Evidence-based optimized perioperative processes have already been described for hip and knee replacements (Ibrahim et al. 2013, Sprowson et al. 2013). These programs can reduce the length of stay to 1–3 days (Kehlet 2013, Khan et al. 2014) without an increased risk of complications or readmission (Glassou et al. 2014), and have also reduced the 2-year mortality rate from 3.8% to 2.7% (Savaridas et al. 2013). However, most studies regarding process optimization for hip and knee replacement were performed in Scandinavia, the UK, and North America, which complicates generalizations to other countries with different healthcare systems. For example, Germany has more hospital beds per capita that Denmark (8 vs. 2.6 beds/1,000 population) (OECD 2017), the hospital reimbursement system is based on diagnosis-related groups, and insured individuals have a legal right to complete a rehabilitation program. Moreover, the median length of stay is 10 days in Germany (IQTIG 2018a, 2018b), versus only 2 days in Denmark (Husted et al. 2016, Knaealloplastikregister 2017). Thus, the setting in Germany can vary considerably from the setting in previous studies that examined optimized care processes, and we are not aware of any similar studies that were performed in Germany. The PROMISE trial is also designed to account for the fact that German patients participate in a rehabilitation program after their hospitalization. Therefore, we hope this prospective multi-center trial will help improve outcomes in the post-hospitalization period by incorporating hospitals that provide different levels of care as well as outpatient and inpatient rehabilitation centers. The resulting information may guide the development of an optimized process that accounts for the patient’s characteristics, expectations, and quality of life, as well as the relevant stress factors, functional outcomes, and economic costs up to 1 year after surgery. The strengths of the study we see in a prospective, multi-centric, intersectoral design, a relatively high number of participants, a very broad data collection at numerous points of time, an above-average follow-up period and an independent external evaluation. Unfortunately, it is not feasible to organize an optimized and a common treatment path at a single center, which intends randomization of the participants to an intervention and control group. We will at least partially address this issue by comparing the results with those from a matched cohort of control patients from a German health insurance database. Parameters that we cannot directly compare between the PROMISE and control groups will be evaluated based on previously published results. Also disadvantageous is that German funding agencies select the rehabilitation facility and we cannot ensure that all participants complete their rehabilitation in PROMISE-affiliated facilities. Although this may reduce the number of participants who complete the full PROMISE process, it will allow us to analyze statistically the benefits of cross-sectoral care.

**Table:** Data collection, time-points, and instruments

Item/score	A	B	C	D	E	F	G
Basic information	X						
Comorbidities **^a^**	X						
Staffelstein	X			X	X		X
Indication	X						
ASA		X					
Preop. anemia assessment		X					
HOOS/KOOS ^a^	X					X	
EQ-5D 5L ^a^	X			X	X	X	
PHQ-4/SSD ^a^	X	X		X	X	X	
OSSS, LOT-R ^a^	X						
HSS or INDICATE knee (preop) ^a^	X						
HSS or INDICATE knee (postop) ^a^						X	
ISAR ^a^		X					
Timed up & go and VAS/NRS		X		X	X		X
Socioeconomic data ^a^	X					X ^b^	
Cost book ^a^				X		X	
Functional goal ^a^						X	
Surgery			X				
Implant details			X				
Functional milestones and discharge				X			
Complications				X			
Rehabilitation					X		
Thrombosis/							
bleeding				X	X		
Follow-up ^a^						X ^b^	
Clinical follow-up							X

A.Indication, 8 weeks preoperatively

B. Preoperative assessment, 1 week preoperatively

C. Operation

D. Hospital stay

E. End of rehabilitation

F. Follow-up at 3, 6, and 12 months postoperatively

G. Clinical follow-up at 12 months postoperatively

**^a^**Patient-reported outcome.

**^b^**Only at the 12-month follow-up

ASA: American Society of Anesthesiologists score,

HOOS: Hip disability and Osteoarthritis Outcome Score,

KOOS: Knee injury and Osteoarthritis Outcome Score,

EQ-5D 5L: EuroQol Group 5-level EQ-5D version,

PHQ-4: Patient Health Questionnaire-4,

SSD: somatic symptom disorders,

OSSS: Online Social Support Scale,

LOT-R: Life Orientation Test-Revised,

HSS: Hospital for Special Surgery,

ISAR: International Society of Arthroplasty Registries,

VAS: visual analogue scale,

NRS: numerical rating scale.

## References

[CIT0001] Beswick A D, Wylde V, Gooberman-Hill R, Blom A, Dieppe P. What proportion of patients report long-term pain after total hip or knee replacement for osteoarthritis? A systematic review of prospective studies in unselected patients. BMJ Open 2012; 2(1): e000435. 10.1136/bmjopen-2011-000435 PMC3289991 22357571

[CIT0002] Conner-Spady B L, Marshall D A, Bohm E, Dunbar M J, Loucks L, Khudairy A A, Noseworthy T W. Reliability and validity of the EQ-5D-5L compared to the EQ-5D-3L in patients with osteoarthritis referred for hip and knee replacement. Qual Life Res 2015; 24(7): 1775–84. 25555837 10.1007/s11136-014-0910-6

[CIT0003] De Oliveira G S, Jr, Castro-Alves L J, Ahmad S, Kendall M C, McCarthy R J. Dexamethasone to prevent postoperative nausea and vomiting: an updated meta-analysis of randomized controlled trials. Anesth Analg 2013; 116(1): 58–74. 23223115 10.1213/ANE.0b013e31826f0a0a

[CIT0004] DRV. Deutsche Rentenversicherung. Reha Therapiestandards Hüft-und Knie-TEP 2016; Available from: http://www.deutsche-rentenversicherung.de/Allgemein/de/Navigation/3_Infos_fuer_Experten/01_Sozialmedizin_Forschung/02_reha_qualitaetssicherung/reha_therapiestandards/indikationen/rts_hueft_knie_tep_node.html. (accessed September 25, 2020).

[CIT0005] Edwards P K, Mears S C, Lowry Barnes C. Preoperative education for hip and knee replacement: never stop learning. Curr Rev Musculoskelet Med 2017; 10(3): 356–64. 28647838 10.1007/s12178-017-9417-4 PMC5577053

[CIT0006] Glassou E N, Pedersen A, Hansen T. Risk of re-admission, reoperation, and mortality within 90 days of total hip and knee arthroplasty in fast-track departments in Denmark from 2005 to 2011. Acta Orthop 2014; 85: 1–8. 10.3109/17453674.2014.942586. 25036718 PMC4164867

[CIT0007] Gronewold J, Dahlmann C, Jäger M, Hermann D M. Identification of hospitalized elderly patients at risk for adverse in-hospital outcomes in a university orthopedics and trauma surgery environment. PLoS One 2017; 12(11): e0187801-e. 29125861 10.1371/journal.pone.0187801 PMC5695284

[CIT0008] Guler O, Mahirogullari M, Isyar M, Piskin A, Yalcin S, Mutlu S, Sahin B. Comparison of quadriceps muscle volume after unilateral total knee arthroplasty with and without tourniquet use. Knee Surg Sports Traumatol Arthrosc 2016; 24(8): 2595–605. 26590567 10.1007/s00167-015-3872-5

[CIT0009] Gylvin S H, Jørgensen C C, Fink-Jensen A, Kehlet H. Psychiatric disease as a risk factor in fast-track hip and knee replacement. Acta Orthop 2016; 87(5): 439–43. 26900724 10.3109/17453674.2016.1151292 PMC5016900

[CIT0010] Hansen T B. Fast track in hip arthroplasty. EFORT Open Rev 2017; 2(5): 179–88. 28630756 10.1302/2058-5241.2.160060 PMC5467651

[CIT0011] Huang Z, Ma J, Shen B, Pei F. General anesthesia: to catheterize or not? A prospective randomized controlled study of patients undergoing total knee arthroplasty. J Arthroplasty 2015; 30(3): 502–6. 25307883 10.1016/j.arth.2014.09.028

[CIT0012] Husted H, Jorgensen C C, Gromov K, Kehlet H. Does BMI influence hospital stay and morbidity after fast-track hip and knee arthroplasty? Acta Orthop 2016; 87(5): 466–72. 27347785 10.1080/17453674.2016.1203477 PMC5016904

[CIT0013] Ibrahim M S, Khan M A, Nizam I, Haddad F S. Peri-operative interventions producing better functional outcomes and enhanced recovery following total hip and knee arthroplasty: an evidence-based review. BMC Med 2013; 11:37-. 23406499 10.1186/1741-7015-11-37 PMC3606483

[CIT0014] IGES-Institut. Infrastruktur und Gesundheit. Weißbuch Gelenkersatz – Versorgungssituation bei endoprothetischen Hüft- und Knieeingriffen in Deutschland 2016. Available from: https://www.iges.com/sites/iges.de/myzms/content/e6666/e13520/e14425/e14438/e14439/attr_objs14757/IGES_PK_Gelenkersatz_Praesentation_Haussler_07062016.pdf_ger.pdf (accessed September 25, 2020).

[CIT0015] IQTIG. Institut für Qualitätssicherung und Transparenz im Gesundheitswesen. Bundesauswertung zum Erfassungsjahr 2017. Hüftendoprothesenversorgung. Qualitätsindikatoren 2018a. Available from: https://www.qs-nrw.org/app/report/doc/bund_2017_hep.pdf. Day of access: September 25, 2020

[CIT0016] IQTIG. Institut für Qualitätssicherung und Transparenz im Gesundheitswesen. Bundesauswertung zum Erfassungsjahr 2017. Knieendoprothesenversorgung.Qualitätsindikatoren 2018b. Available from: https://iqtig.org/downloads/auswertung/2017/kep/QSKH_KEP_2017_BUAW_V02_2018-08-01.pdf (accessed September 25, 2020).

[CIT0017] Kehlet H. Fast-track hip and knee arthroplasty. Lancet 2013; 381(9878): 1600–2. doi:(13)61003-X. 23663938 10.1016/S0140-6736(13)61003-X

[CIT0018] Kelly E G, Cashman JP, Imran F H, Conroy R, O’Byrne J. Systematic review and meta-analysis of closed suction drainage versus non-drainage in primary hip arthroplasty. Surg Technol Int 2014; 24: 295–301. 24574017

[CIT0019] Khan S K, Malviya A, Muller S D, Carluke I, Partington P F, Emmerson K P, Reed M R. Reduced short-term complications and mortality following Enhanced Recovery primary hip and knee arthroplasty: results from 6,000 consecutive procedures. Acta Orthop 2014; 85(1): 26–31. 24359028 10.3109/17453674.2013.874925 PMC3940988

[CIT0020] Klässbo M, Larsson E, Mannevik E. Hip disability and osteoarthritis outcome score: an extension of the Western Ontario and McMaster Universities Osteoarthritis Index. Scand J Rheumatol 2003; 32(1): 46–51. 12635946 10.1080/03009740310000409

[CIT0021] Knaealloplastikregister D. Årsrapport 2017; 2017. Available from: 2020https://www.sundhed.dk/content/cms/99/4699_dkr-aarsrapport-2019_til-offentliggoerelse.pdf (accessed September 25, 2020).

[CIT0022] Ljungqvist O. Jonathan E. Rhodes lecture 2011: Insulin resistance and enhanced recovery after surgery. JPEN J Parenter Enteral Nutr 2012; 36(4): 389–98. 22577121 10.1177/0148607112445580

[CIT0023] Ljungqvist O, Scott M, Fearon K C. Enhanced recovery after surgery: a review. JAMA Surg 2017; 152(3): 292–8. 28097305 10.1001/jamasurg.2016.4952

[CIT0024] Mayring P. Qualitative Inhaltsanalyse. Grundlagen und Techniken Weinheim: Deutscher Studien Verlag; 2000.

[CIT0025] McLean E, Cogswell M, Egli I, Wojdyla D, de Benoist B. Worldwide prevalence of anaemia, WHO Vitamin and Mineral Nutrition Information System, 1993–2005. Public Health Nutr 2009; 12(4): 444–54. 18498676 10.1017/S1368980008002401

[CIT0026] Nielsen C S, Jans Ø, Ørsnes T, Foss N B, Troelsen A, Husted H. combined intra-articular and intravenous tranexamic acid reduces blood loss in total knee arthroplasty: a randomized, double-blind, placebo-controlled trial. J Bone Joint Surg Am 2016; 98(10): 835–41. 27194493 10.2106/JBJS.15.00810

[CIT0027] OECD. Hospital beds 2017. Available from: https://data.oecd.org/healtheqt/hospital-beds.htm (accessed September 25, 2020).

[CIT0028] Okamoto T, Ridley R J, Edmondston S J, Visser M, Headford J, Yates P J. Day-of-surgery mobilization reduces the length of stay after elective hip arthroplasty. J Arthroplasty 2016; 31(10): 2227–30. 27209333 10.1016/j.arth.2016.03.066

[CIT0029] Paulsen A, Roos E M, Pedersen A B, Overgaard S. Minimal clinically important improvement (MCII) and patient-acceptable symptom state (PASS) in total hip arthroplasty (THA) patients 1 year postoperatively. Acta Orthop 2014; 85(1): 39–48. 24286564 10.3109/17453674.2013.867782 PMC3940990

[CIT0030] Roos E M, Toksvig-Larsen S. Knee injury and Osteoarthritis Outcome Score (KOOS): validation and comparison to the WOMAC in total knee replacement. Health Qual Life Outcomes 2003; 1: 17. 12801417 10.1186/1477-7525-1-17 PMC161802

[CIT0031] Savaridas T, Serrano-Pedraza I, Khan S K, Martin K, Malviya A, Reed M R. Reduced medium-term mortality following primary total hip and knee arthroplasty with an enhanced recovery program: a study of 4,500 consecutive procedures. Acta Orthop 2013; 84(1): 40–3. 23368747 10.3109/17453674.2013.771298 PMC3584601

[CIT0032] Schmitt J, Lange T, Günther K P, Kopkow C, Rataj E, Apfelbacher C, Aringer M, Böhle E, Bork H, Dreinhöfer K, Friederich N, Frosch K H, Gravius S, Gromnica-Ihle E, Heller K D, Kirschner S, Kladny B, Kohlhof H, Kremer M, Leuchten N, Lippmann M, Malzahn J, Meyer H, Sabatowski R, Scharf H P, Stoeve J, Wagner R, Lützner J. Indication criteria for total knee arthroplasty in patients with osteoarthritis: a multi-perspective consensus study. Z Orthop Unfall 2017; 155(5): 539–48. 29050054 10.1055/s-0043-115120

[CIT0033] Sharma S, Iorio R, Specht L M, Davies-Lepie S, Healy W L. Complications of femoral nerve block for total knee arthroplasty. Clin Orthop Relat Res 2010; 468(1): 135–40. 19680735 10.1007/s11999-009-1025-1 PMC2795813

[CIT0034] Smith I, Kranke P, Murat I, Smith A, O’Sullivan G, Søreide E, Spies C, in’t Veld B. Perioperative fasting in adults and children: guidelines from the European Society of Anaesthesiology. Eur J Anaesthesiol 2011; 28(8): 556–69. 21712716 10.1097/EJA.0b013e3283495ba1

[CIT0035] Sozialgesetzbuch V 2020. Fünftes Buch (SGB V), Gesetzliche Krankenversicherung. Available from: https://www.sozialgesetzbuch-sgb.de/sgbv/110a.html (accessed October 12, 2020).

[CIT0036] Sprowson A, McNamara I, Manktelow A. (v) Enhanced recovery pathway in hip and knee arthroplasty: “fast track” rehabilitation. Orthop Trauma 2013; 27(5): 296–302. doi:.

[CIT0037] Vaglio S, Prisco D, Biancofiore G, Rafanelli D, Antonioli P, Lisanti M, Andreani L, Basso L, Velati C, Grazzini G, Liumbruno G M. Recommendations for the implementation of a Patient Blood Management programme. Application to elective major orthopaedic surgery in adults. Blood Transfus 2016; 14(1): 23–65. 26710356 10.2450/2015.0172-15 PMC4731340

[CIT0038] Wainwright T, Gill M, McDonald D, Middleton R, Reed M, Sahota O, Yates P, Ljungqvist O. Consensus statement for perioperative care in total hip replacement and total knee replacement surgery: Enhanced Recovery After Surgery (ERAS®) Society recommendations. Acta Orthop 2019; 91 (1): 3–19. 31663402 10.1080/17453674.2019.1683790 PMC7006728

[CIT0039] Yun X D, Yin X L, Jiang J, Teng Y J, Dong H T, An L P, Xia Y Y. Local infiltration analgesia versus femoral nerve block in total knee arthroplasty: a meta-analysis. Orthop Traumatol Surg Res 2015; 101(5): 565–9. 25987449 10.1016/j.otsr.2015.03.015

